# Development of a Combined Oxidative Stress and Endoplasmic Reticulum Stress-Related Prognostic Signature for Hepatocellular Carcinoma

**DOI:** 10.2174/0113862073257308231026073951

**Published:** 2023-11-10

**Authors:** Hui Ma, Zhongchen Li, Rongxin Chen, Zhenggang Ren

**Affiliations:** 1 Liver Cancer Institute, Zhongshan Hospital, Fudan University, Shanghai, China;; 2 Rutgers Cancer Institute of New Jersey, New Brunswick, New Jersey, USA

**Keywords:** Hepatocellular carcinoma, oxidative stress, endoplasmic reticulum stress, prognosis, OESGs, ER

## Abstract

**Background:**

Oxidative stress and endoplasmic reticulum stress are important components of the cellular stress process, which plays a critical role in tumor initiation and progression.

**Methods:**

First, the correlation between oxidative stress and endoplasmic reticulum stress was detected in 68 human hepatocellular carcinoma (HCC) tissue microarray samples by immunohistochemistry. Differentially expressed oxidative stress- and endoplasmic reticulum stress-related genes (OESGs) then were screened in HCC. Next, an OESGs prognostic signature was constructed for HCC in the training cohort (TCGA-LIHC from The Cancer Genome Atlas), by least absolute shrinkage and selection operator Cox and stepwise Cox regression analyses, and was verified in the external cohort (GSE14520 from the Gene Expression Omnibus). The MCP counter was employed to evaluate immune cell infiltration. The C-index was used to evaluate the predictive power of prognostic signature. Finally, a prognostic nomogram model was constructed to predict the survival probability of patients with HCC based on the results of Cox regression analysis.

**Results:**

We demonstrated a positive correlation between oxidative stress and endoplasmic reticulum stress in human HCC samples. We then identified five OESGs as a prognostic signature consisting of *IL18RAP*, *ECT2*, *PPARGC1A*, *STC2,* and *NQO1* for HCC. Related risk scores correlated with tumor stage, grade, and response to transcatheter arterial chemoembolization therapy, and the higher risk score group had less T cells, CD8^+^ T cells, cytotoxic lymphocytes and natural killer cell infiltration. The C-index of our OESGs prognostic signature was superior to four previously published signatures. Furthermore, we developed a nomogram based on the OESGs prognostic signature and clinical parameters for patients with HCC that is an effective quantitative analysis tool to predict patient survival.

**Conclusion:**

The OESGs signature showed excellent performance in predicting survival and therapeutic responses for patients with HCC.

## 1 INTRODUCTION

Hepatocellular carcinoma (HCC), the major primary liver cancer, is the sixth most common cancer and the fourth leading cause of cancer-related deaths worldwide [1]. HCC is usually diagnosed at advanced stages, resulting in limited treatment options [2]. Genomic analyses have revealed substantial differences in the molecular characteristics of HCC cells and their tumor microenvironment even in samples with the same clinical grade [2, 3]. The variation in molecular typing of patients with HCC also leads to differences in treatment efficacy and prognosis [4]. The prognostic biomarkers commonly used in clinical practice are still not satisfactory owing to the heterogeneous nature of HCC [2]. Therefore, it is necessary to create new therapeutic and prognostic models for HCC.

Oxidative stress occurs as a result of an imbalance between the generation of reactive oxygen species and antioxidant molecules in cells, causing damage to DNA, lipids, and proteins [5]. HCC can develop from a background of chronic viral hepatitis, including from infection by hepatitis B or hepatitis C viruses, nonalcoholic steatohepatitis, or cirrhosis; these diseases enhance the production of reactive oxygen species and induction of oxidative stress in tumor cells and their microenvironment [5]. And oxidative stress contributes to HCC tumorigenesis and progression in multiple pathways [5, 6].

The endoplasmic reticulum (ER) is the largest organelle in eukaryotic cells and is involved in protein synthesis and transportation, protein folding, lipid and steroid synthesis, carbohydrate metabolism, and calcium storage [7]. Pathological or physiological stress can interfere with the normal protein folding processes of the ER; the resulting accumulation of unfolded or misfolded proteins in the ER lumen leads to ER stress [7]. Hepatocytes are enriched with ER and are susceptible to ER stress, which participates in the development of aggressive and drug-resistant HCC [8].

Although the association between oxidative stress and ER stress remains to be fully elucidated, these two cellular stresses are closely linked in tumors [9]. Redox homeostasis is critical for the protein-folding process and disulfide bond formation in ER [7]. Alterations in ER-mediated protein-folding pathways can enhance reactive oxygen species production and cause an imbalance in reactive oxygen species, disturbing both ER and redox homeostasis [9]. Oxidative stress and ER stress are intrinsically linked, as one process may trigger the other in various scenarios encountered by the tumor cells [9, 10].

Herein, we demonstrated a positive correlation between oxidative stress and ER stress in human HCC samples. We used a systematic integrative analysis of oxidative stress- and ER stress-related genes (OESGs) to construct a prognostic signature for HCC [11], and the prognostic value of the signature was analyzed. Moreover, a prognostic nomogram was developed to provide a quantitative analysis tool to predict prognostic risk in patients with HCC.

## 2 MATERIALS AND METHODS

### 2.1 Human HCC Specimens

Microarrays of tissue samples from 68 patients with histologically confirmed HCC were purchased from BioCoreUSA (DLV809a) [12].

### 2.2 Immunohistochemistry

Pathological slides were stained according to standard procedures. Briefly, after incubation with anti-8-hydroxy-2’-deoxyguanosine (8-OHdG) antibody (1:1000; ab48508, Abcam) and anti-BiP antibody (1:400, 3177, Cell Signaling Technology) at 4 °C overnight, the slides were incubated with appropriate secondary antibodies. Images were taken under a light microscope; three fields were selected at random for uniform image capture. Staining intensity (0, 1+, 2+, or 3+) was determined for each cell in a fixed field, and the H-score was assigned using the following formula: [1 × (% cells 1+) + 2 × (% cells 2+) + 3 × (% cells 3+)] [13].

### 2.3 Data Acquisition and Identification of OESGs

This study included mRNA expression data and clinical features of patients with HCC from three publicly available data sets: The Cancer Genome Atlas Liver Hepatocellular Carcinoma data collection (TCGA-LIHC), and GSE14520 [14] and GSE104580 from the Gene Expression Omnibus. Genes set associated with oxidative stress and ER stress were acquired from the Molecular Signatures Database [15] (http://www.gsea-msigdb.org/gsea/msigdb), and the two gene sets were deduplicated and combined for subsequent analysis.

### 2.4 Nonnegative Matrix Factorization (NMF] Clustering Algorithm

The R package limma [16] (version 3.10.3,http://www.bioconductor.org/packages/2.9/bioc/html/limma.html) was used to analyze differentially expressed genes (DEGs) in HCC and para-carcinoma liver samples. The absolute values of log2 (fold change) greater than 1 and adjusted *p* values less than 0.05 were used to include DEGs, from which the differentially expressed OESGs were extracted. HCC samples from TCGA-LIHC were grouped using the R package ConsensusClusterPlus [17] (version 1.54.0, http://www.bioconductor.org/packages/release/bioc/html/ConsensusClusterPlus.html). The number of clusters (*K*) ranged from 2 to 6, and cophenetic, dispersion and profile measures were used to determine the ideal number of clusters.

### 2.5 Construction and Validation of the OESGs Prognostic Signature

The univariate Cox regression analysis was performed to identify prognosis-related DEGs in the TCGA-LIHC data set using the R package gwasurvivr [18] (version1.16.0, https://bioconductor.org/packages/gwasurvivr/). Least absolute shrinkage and selection operator (LASSO) Cox and stepwise Cox regression analyses were performed to survey the key prognosis-related qualities and to set up prognostic characteristics using the R package glmnet [19] (version 1.2, https://cran.r-project.org/web/packages/glmnet/index.html) and survminer (version 0.4.9, https://cran.rstudio.com/web/packages/survminer/index.html). The risk score was calculated based on gene expression levels and corresponding coefficient values. We stratified patients into two subgroups, according to the median risk score (RS). The effectiveness of the prognostic signature to assess survival, and measure risk were evaluated by and Cox models of relative risk, respectively. The receiver operating characteristic (ROC) curve, Kaplan-Meier survival, and C-index were performed using the R package gwasurvivr.

### 2.6 Immune cell Infiltration Analysis

The MCP counter [20] (http://github.com/ebecht/MCPcounter) was used to explore the proportion of different types of immune cells in the tumor microenvironment of HCC samples. The tertiary lymphoid structure (TLS) score was calculated using the R package GSVA (version 1.36.2, http://bioconductor.org/packages/release/bioc/html/GSVA.html) in accordance with the expression of the TLS signature genes (*CCR6*, *CD1D*, *CD79B*, *CETP*, *EIF1AY*, *LAT*, *PTGDS*, *RBP5* and *SKAP1*) [21]. The immune cytolytic activity (CYT) score, was assessed by calculating the average of *GZMA* and *PRF1* expression [22].

### 2.7 Construction of a Prognostic Nomogram

Univariate and multivariate Cox regression analyses were used to determine independent prognostic characteristics of patients with HCC in the TCGA-LIHC cohort, and a prognostic nomogram model was constructed to predict the survival probability of patients with HCC based on the results of Cox regression analysis. The calibration graph was generated to show the differences between predicted and actual survival rates of patients with HCC using R package rms (version 5.1-2, https://cran.r-project.org/web/packages/rms/index.html).

### 2.8 Statistical Analysis

All statistical analyses were performed with R software (version 4.0.3) or GraphPad Prism (version 7.0), setting statistical significance at *p* <0.05. Comparisons were assessed using unpaired Student’s *t*-tests, Pearson *χ^2^* tests or Fisher’s exact tests, as appropriate. Correlations were evaluated using Pearson’s *r*. For Kaplan-Meier survival curves, statistical significance was estimated with the log-rank test.

## 3 RESULTS

### 3.1 A positive Correlation between Oxidative Stress and ER Stress in Human HCC Tissues

**Fig. (1):**
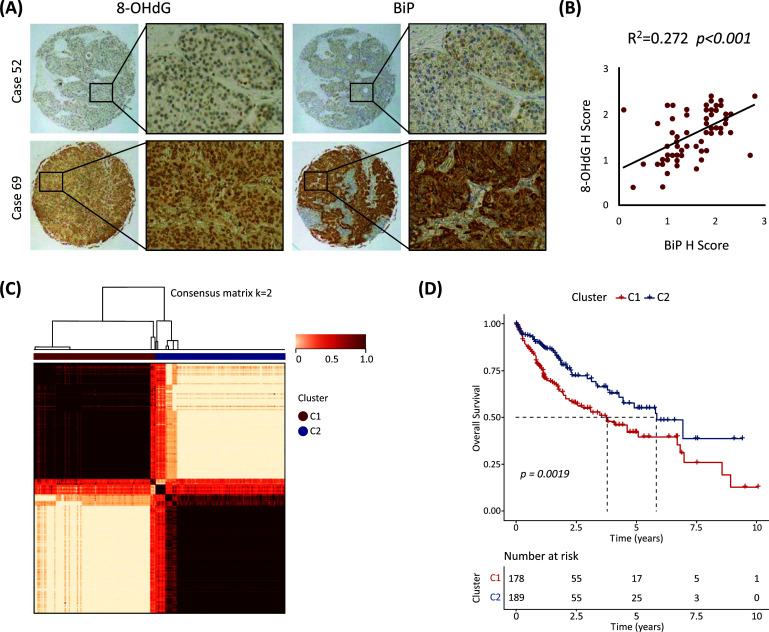
Identification of OESGs clusters based on an NMF algorithm. (**A**) Representative immunohistochemistry images showing low expression (case 52) and high expression (case 69) of 8-OHdG and BiP. (**B**) Pearson correlation analysis between 8-OHdG and BiP levels in tumor specimens from 68 patients with HCC. (**C**) Heatmap of HCC subtypes in TCGA-LIHC based on OESGs using the NMF clustering algorithm with consensus matrix *K*=2. (**D**) Kaplan-Meier curve of overall survival for two HCC subtypes. The overall survival of cluster 1 was significantly worse than that of cluster 2.

Immunohistochemistry was performed on tissue microarray samples from tumor specimens of 68 patients with HCC to quantify 8-OHdG and BiP levels, markers of oxidative stress and ER stress, respectively [7, 12]. Fig. (**1A**) shows representative images of low expression (case 52) and high expression (case 69). The H-score was used to denote the level of target expression, and statistical analysis revealed a positive correlation between 8-OHdG and BiP levels (R^2^=0.272; *p* <0.001; Fig. **1B**).

### 3.2 Identification of OESGs Clusters based on an NMF Algorithm

Given the evidence of the interaction between oxidative stress and ER stress in HCC tissues, an OESGs signature was constructed. After filtering and deduplication, 975 OESGs were included (Table **S1**). Further difference analysis identified 185 DEGs from OESGs in HCC samples compared with the para-carcinoma liver samples, with 61 DEG upregulation and 124 DEGs downregulated (Table **S2**). Based on the DEGs, patients with HCC were classified into two OESGs subtypes (clusters 1 and 2) using the NMF clustering algorithm (Fig. **1C**), and the overall survival of cluster 1 was significantly worse than that of cluster 2 (*p*=0.0019; Fig. **1D**). The distribution of clinical characteristics between the two OESGs clusters was also compared. The proportion of patients with high-grade and late-stage HCC in cluster 1 was higher than that in cluster 2 (Table **S3**).

### 3.3 Construction and Validation of the OESGs Prognostic Signature

**Fig. (2):**
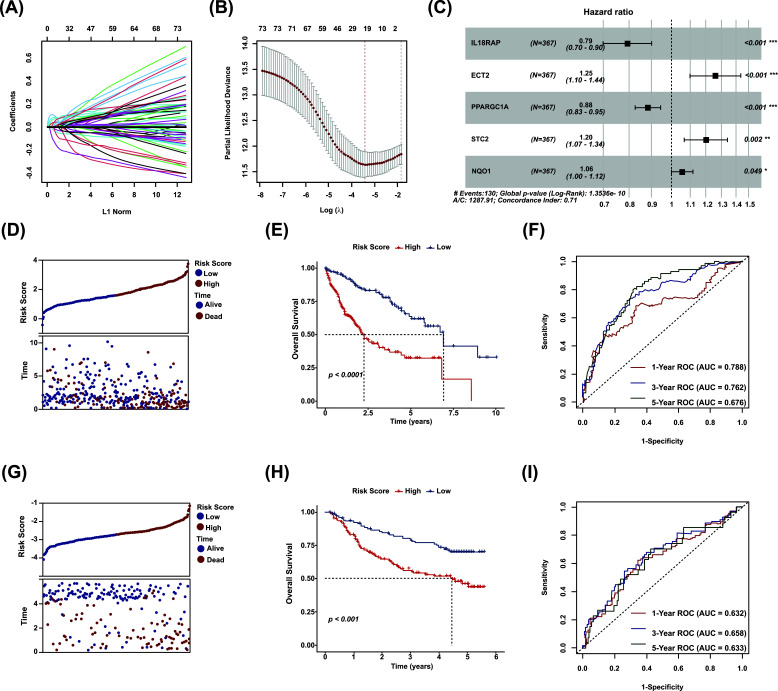
Construction and validation of the OESGs prognostic signature. (**A**) Coefficients of independent variables in LASSO regression. (**B**) Cross-validation of parameter selection in LASSO regression. (**C**) Forest plot of the OESGs model by stepwise Cox regression algorithm. RS = - 0.2310 × *IL18RAP* + 0.2303 × *ECT2* - 0.1228 × *PPARGC1A* + 0.1809 × *SCT2* + 0.0567 × *NQO1*. (**D**) Distribution of risk score and survival status in the internal (TCGA-LIHC) cohort. (**E**) Kaplan-Meier curve analyses for the high-risk and low-risk groups in the internal cohort. (**F**) The ROC curves of the OESGs prognostic signature at 1, 3, and 5 years in the internal cohort. (**G**) Distribution of risk score and survival status in the external (GSE14520) cohort. (**H**) Kaplan-Meier curve analyses for the high-risk and low-risk groups in the external cohort. (**I**) The ROC curves of the OESGs prognostic signature at 1, 3, and 5 years in the external cohort.

We identified prognosis-related OESGs in the TCGA-LIHC cohort using the univariate Cox regression analysis and constructed a prognostic prediction model based on the prognosis-related OESGs using LASSO Cox regression analysis. The coefficients of independent variables in LASSO regression are shown in Fig. (**2A**). We identified 19 genes of interest based on the optimal log value of lambda (Fig. **2B**). By stepwise Cox regression method, an RS that represented the comprehensive index of oxidative stress and ER stress status for the OESGs signature was calculated according to expression levels of five genes and corresponding coefficients, as follows: RS = - 0.2310 × *IL18RAP* + 0.2303 × *ECT2* - 0.1228 × *PPARGC1A* + 0.1809 × *SCT2* + 0.0567 × *NQO1*. A forest plot showed that the five genes in the risk model were closely related to prognosis (Fig. **2C**). RSs for HCC patients in the TCGA-LIHC cohort were calculated, and based on the median RS, patients were divided into high- and low-risk groups. A scatter plot depicted the distribution of RSs and their relationship to survival outcomes (Fig. **2D**). Kaplan-Meier survival analysis revealed that patients with high RSs had a poor prognosis (Fig. **2E**). ROC analysis revealed that the OESGs signature had a good prognostic performance with AUCs at 1, 3, and 5 years of 0.788, 0.762, and 0.676, respectively (Fig. **2F**).

To further assess the robustness and predictive ability of the OESGs signature, external validation was performed using the GSE14520 cohort. The RSs of patients were calculated with the same formula, and patients were classified into the high- and low-risk subgroups in the validation cohort. The distribution of the RSs and their associations with survival status is illustrated in Fig. (**2G)**. Kaplan-Meier survival and ROC analysis were performed. Patients in the high-risk subgroup were prominently relevant to short overall survival (Fig. **2H**) and the AUCs of 1-, 3-, and 5-year survival are shown in Fig. (**2I**).

### 3.4 Correlation of the OESGs Prognostic Signature with Clinical Features and Response to Therapy

**Fig. (3):**
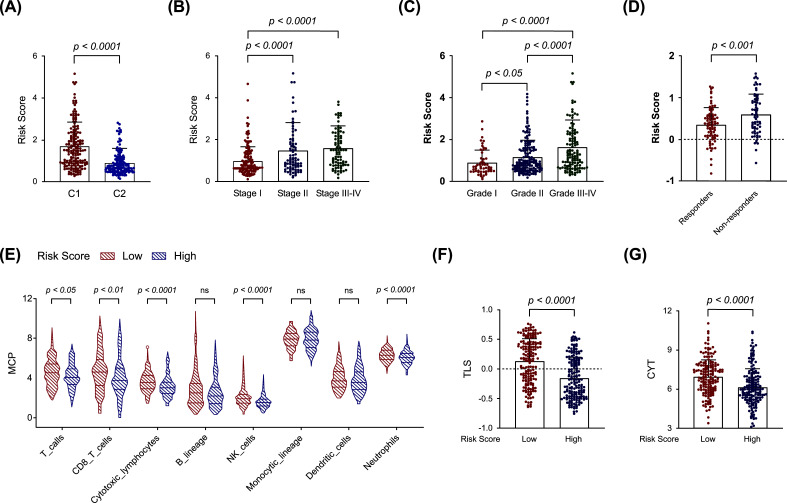
Correlation of the OESGs prognostic signature with clinical features and response to therapy. The difference in risk score between different clinical features of (**A**) cluster molecular subtypes, (**B**) tumor stage, and (**C**) tumor grade. (**D**) The difference in risk score between responders and non-responders to transcatheter arterial chemoembolization therapy. (**E**) Comparison of the difference in immune cell infiltration between the high-risk and low-risk groups. (**F**) Correlation of the risk score and TLS score. (**G**) Correlation of the risk score and CYT score.

To investigate whether the OESGs signature correlated with clinical features and response to antitumor therapy, RSs of different subgroups were compared in TCGA-LIHC and GSE104580 cohorts. The RS of OESGs cluster 1 with a poor prognosis was higher than that of cluster 2 (Fig. **3A**). Patients with advanced HCC had a higher RS than those with early-stage HCC (Fig. **3B**), and patients with higher HCC pathological grade had significantly increased RS (Fig. **3C**). As the most frequently mutated genes in HCC [23], *TP53* mutation frequency was significantly higher in the high RS group, while *TERT* mutation frequency showed no significant differences (Fig. **S1**). Additionally, the group with no response to transcatheter arterial chemoembolization therapy, recommended as the first-line treatment for patients at the intermediate HCC stage, had an increased RS (Fig. **3D**). To further investigate the potential application value of the OESGs prognostic signature in immunotherapy efficacy, the immune cell infiltration was analyzed. As shown in Fig. (**3E**), the high RS group had less T cells, CD8^+^ T cells, cytotoxic lymphocyte, natural killer cells and neutrophil infiltration. Notably, the high RS group also had lower TLS and CYT scores (Fig. **3F** and **3G**), well-established biomarkers for predicting immune efficacy. These findings indicate that the OESGs prognostic signature shows good prognostic predictive power in accordance with different clinical characteristics and different responses to antitumor therapies.

### 3.5 Comparison of the OESGs Prognostic Signature with Published Signatures

**Fig. (4):**
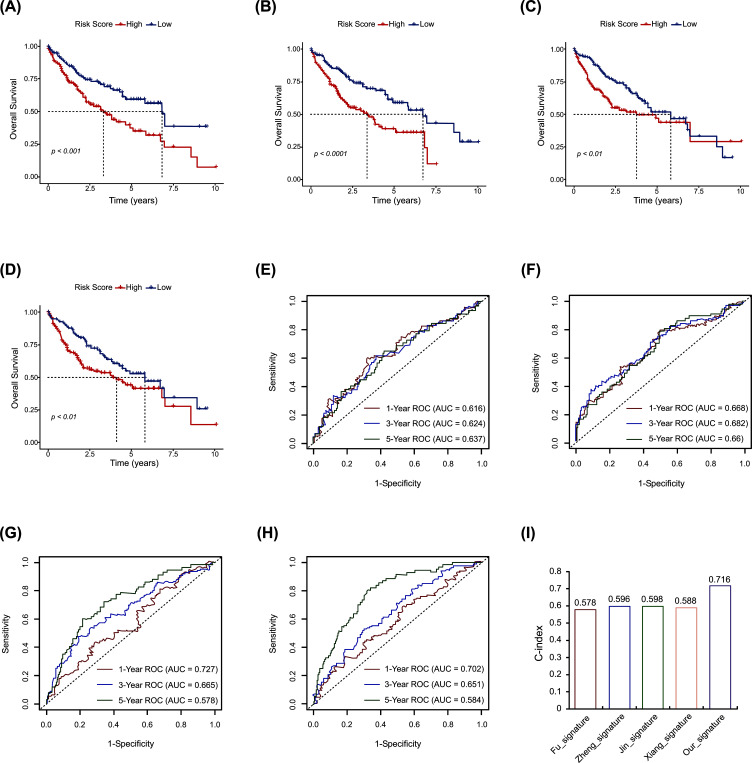
Comparison of the OESGs prognostic signature with the published signatures. Kaplan-Meier curve of the signatures established by (**A**) Fu *et al*., (**B**) Zheng *et al*., (**C**) Jin *et al*., and (**D**) Xiang *et al*.. ROC curve of (**E**) the Fu signature, (**F**) the Zheng signature, (**G**) the Jin signature, and (**H**) the Xiang signature. (**I**) Comparison of C-indexes for the four published signatures and our OESGs signature.

To explore whether our OESGs signature had a superior predictive ability, we compared it with four published prognostic models, the Fu signature [24], the Zheng signature [25], the Jin signature [26], and the Xiang signature [27]. Using the same method to calculate the RSs, these four signatures were able to categorize the TCGA-LIHC cohort into a high-risk subgroup and a low-risk subgroup that had significantly different outcomes respectively (Fig. **4A**-**D**). ROC curve analysis found that the AUCs of our model were higher than those of the four published signatures (Fig. **2F**, and Fig. **4E**-**4H**). The C-index, used to evaluate the predictive power of models, was the highest in our OESGs signature, followed by the other four published ones (Fig. **4I**). These findings highlight the consistently superior performance of our OESGs prognostic signature compared with the four published signatures.

### 3.6 Construction of a Nomogram Based on the OESGs Prognostic Signature

**Fig. (5):**
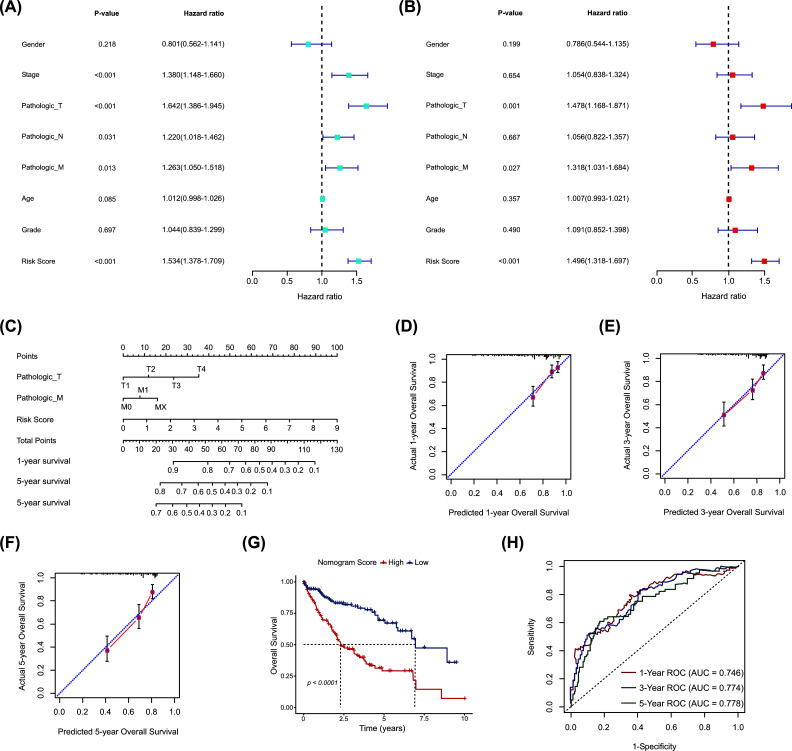
Construction of a nomogram based on the OESGs prognostic signature. (**A**) Univariate Cox regression and (**B**) multivariate Cox regression analyses of overall survival in TCGA-LIHC cohort. (**C**) Nomogram for predicting the overall survival in TCGA-LIHC cohort at 1, 3, and 5 years. Calibration curve for consistency between (**D**) 1-year, (**E**) 3-year, and (**F**) 5-year nomogram-predicted survival and actual survival. (**G**) Kaplan-Meier curve of overall survival for high and low nomogram score groups. (**H**) ROC curves of nomograms for 1-year, 3-year, and 5-year survival.

Given that a comprehensive analysis of HCC patients could better predict clinical outcomes, we further performed Cox regression analyses with the OESGs RS, clinical information, and patient prognosis in the TCGA-LIHC cohort. OESGs RS was found to be correlated with prognosis significantly in both univariate [hazard ratio (95% CI) = 1.534 (1.376-1.709), *p* <0.001] (Fig. **5A**) and multivariate regression analyses [hazard ratio (95% CI) = 1.496 (1.318-1.697), *p* <0.001] (Fig. **5B**), suggesting that the OESGs prognostic signature had a good clinical predictive value independently. Three variables with a *p-*value less than 0.05 were determined by multiple regression, namely, T stage, M stage and RS, and a nomogram model was established to predict survival risk for patients with HCC (Fig. **5C**). As shown in Fig. **5D**-**5F**, the overlap between the forecasted and actual probabilities of 1-, 3-, and 5-year survival rates in calibration curves indicated good agreement. Moreover, HCC samples with a high nomogram score had a significantly poorer prognosis than those with a low nomogram score (Fig. **5G**). The AUCs of the prognostic model reached 0.746, 0.774, and 0.778 at 1, 3, and 5 years, respectively (Fig. **5H**), which confirms the good prognostic value of our model.

## 4 DISCUSSION

In the past few decades, considerable progress has been made towards understanding the risk factors and molecular characteristics of HCC [2, 3]. However, the existing prognostic staging system has many limitations and needs to be improved using gene sequencing technology so that patients can benefit from the development of more targeted treatments and the ability to predict clinical outcomes more accurately [2, 3]. There is increasing evidence that the cellular stress status of tumors can substantially affect HCC development and may be related to advanced disease and adverse clinical outcomes [28, 29]; hence, new therapeutic and prognostic models based on cellular stress-related genes show promise to assist in effective management of HCC.

Oxidative stress and ER stress, the two well-known cellular stresses in HCC tumors and their microenvironment are closely linked with and promoted by each other [9]. In this study, we confirmed a positive correlation between oxidative stress and ER stress in human HCC tissue samples (R^2^=0.272; *p* <0.001; Fig. **1B**). We identified five OESGs as a prognostic signature consisting of *IL18RAP*, *ECT2*, *PPARGC1A*, *STC2* and *NQO1* for HCC in the training cohort TCGA-LIHC, by LASSO Cox and stepwise Cox regression analyses (Fig. **2A**-**F**), and the OESGs prognostic signature was verified in external cohort GSE14520 (Fig. **2G**-**I**). Related risk scores correlated with tumor stage (Fig. **3B**), grade (Fig. **3C**), and response to transcatheter arterial chemoembolization therapy (Fig. **3D**); the higher risk score group had less T cell, CD8^+^ T cell, cytotoxic lymphocyte and natural killer cell infiltration (Fig. **3E**-**G**). We also developed a nomogram based on the OESGs prognostic signature and clinical parameters for patients with HCC that is an effective quantitative analysis tool to predict patient survival (Fig. **4A**-**H**).

Interleukin 18 receptor accessory protein (IL18RAP) could enhance the IL-18-binding activity of the IL-18 receptor and play a role in IL-18 signaling, which regulates the cross-talk between inflammation, oxidative stress, and ER stress [30]. IL18RAP is associated with HCC prognosis and is important for immune cell infiltration into the tumor microenvironment [31]. Epithelial cell transforming 2 (ECT2) is expressed in a cell cycle-dependent manner during liver regeneration and is upregulated after DNA damage caused by oxidative stress [32]. ECT2 is a biomarker that can be used for diagnosis and prognostic prediction in HCC, and its overexpression could promote lactic acid production through the ECT2/PLK1/PTEN pathway, enhance tumor-associated macrophage infiltration, and suppress immune cell function [33]. PPARG coactivator 1 alpha (PPARGC1A, also named PGC1α) is a transcriptional coactivator that regulates the genes involved in energy metabolism; it provides a direct link between external physiological stimuli and the regulation of mitochondrial biogenesis [34]. Low levels of PPARGC1A expression indicate a poor prognosis for patients with HCC [35]. Stanniocalcin 2 (STC2) is a glycoprotein that plays a role in the regulation of cell metabolism and cellular calcium and phosphate homeostasis [36]. STC2 is substantially stimulated under stress conditions such as ER stress, hypoxia, and nutrient deprivation; the overexpression of STC2 is positively correlated with tumor growth, invasion, metastasis, and poor patient prognosis, highlighting its potential as a biomarker [37]. NAD(P)H dehydrogenase quinone 1 (NQO1) is a cytosolic detoxifying enzyme that maintains intracellular redox homeostasis by catalyzing quinones to hydroquinones with NADH and NADPH as electron donors [38]. NQO1 is downstream of the NRF2 pathway, which is involved in signaling in response to oxidative stress and ER stress [6, 7]. Elevated NQO1 levels are associated with metastasis and poor prognosis in patients with HCC [39].

In recent years, both oxidative stress and ER stress have been associated with antitumor immunity [6, 7]. Oxidative stress can cause oxidative DNA damage, which is an important initiator of malignant tumors. Oxidative DNA damage can remodel the tumor immune microenvironment through the cGAS-STING pathway, which improves tumor response to PD-1 antibody treatment [12]. ER stress initiates the unfolded protein response through three ER transmembrane proteins: activating transcription factor 6, inositol-requiring enzyme 1α, and PRKR-like ER kinase [40]. All three ER stress-response proteins and their related pathways influence diverse tumorigenic and immune-regulatory programs that dictate malignant progression, antitumor immunity, and response to treatment [7, 41]. These findings provide a mechanistic explanation for our results. However, the specific mechanism of cross-talk between oxidative stress and ER stress in the immune environment of HCC still needs to be further explored.

There are certain limitations of this study. First, clinical information related to surgery and tumor markers was not considered. Second, we established the OESGs prognostic signature and the nomogram based on public databases, which need to be tested in large multicenter prospective cohorts.

## CONCLUSION

We created a novel prognostic signature based on the integrated analysis of OESGs that has satisfactory efficiency in predicting the prognosis of patients with HCC. We also developed a nomogram for patients with HCC that is an effective quantitative analysis tool and could contribute to precise and personalized HCC treatment.

## Data Availability

Publicly available datasets were analyzed in this study. The names of the repositories and accession numbers can be found in the article.

## LIST OF ABBREVIATIONS

8-OHdG: 8-hydroxy-2’-deoxyguanosine
AUC: Area Under Curve
cGAS: cyclic GMP-AMP Synthase
CYT: Cytolytic
DEG: Differentially Expressed Gene
ECT2: Epithelial Cell Transforming 2
ER: Endoplasmic Reticulum
HCC: Hepatocellular Carcinoma
IL18RAP: Interleukin 18 Receptor Accessory Protein
LASSO: Least Absolute Shrinkage and Selection Operator
LIHC: Liver Hepatocellular Carcinoma
NMF: Nonnegative Matrix Factorization
NQO1: NAD(P)H Dehydrogenase Quinone 1
OESG: Oxidative Stress- and Endoplasmic Reticulum Stress-related Gene
PPARGC1A: PPARG coactivator 1 alpha
ROC: Receiver Operating Characteristic
RS: Risk Score
STC2: Stanniocalcin 2
STING: Stimulator of Interferon Genes
TCGA: The Cancer Genome Atlas
