# Supplementary material for: Development of a Combined Oxidative Stress and Endoplasmic Reticulum Stress-Related Prognostic Signature for Hepatocellular Carcinoma
Source: Comb Chem High Throughput Screen. 2023 Nov 10;27(19):2850–60. doi: 10.2174/0113862073257308231026073951 (PMC11497145; doi:10.2174/0113862073257308231026073951)
Supplement: Supplementary file 1 [file CCHTS-27-2850_SD1.pdf]

Supplementary Material

Development of a Combined Oxidative Stress and Endoplasmic Reticulum Stress-Related Prognostic Signature for Hepatocellular Carcinoma

Hui Ma[1,2*], Zhongchen Li[1], Rongxin Chen[1] and Zhenggang Ren[1]

[1]*Liver Cancer Institute, Zhongshan Hospital, Fudan University, Shanghai, China;* [2]*Rutgers Cancer Institute of New Jersey, New Brunswick, New Jersey, USA*

Table S3. Clinical characteristics of the two HCC subtypes identified based on OESGs.

Characteristics	C1 (n=178)	C2 (n=189)	Total (n=367)	*p* value
Gender				$p < 0.001$
Female	75(20.44%)	44(11.99%)	119(32.43%)	
Male	103(28.07%)	145(39.51%)	248(67.57%)	
Stage				$p < 0.05$
I	68(18.53%)	103(28.07%)	171(46.59%)	
II	44(11.99%)	41(11.17%)	85(23.16%)	
III	51(13.90%)	32(8.72%)	83(22.62%)	
IV	2(0.54%)	2(0.54%)	4(1.09%)	
NA	13(3.54%)	11(3.00%)	24(6.54%)	
Pathologic_T				$p < 0.01$
NA	0(0%)	2(0.54%)	2(0.54%)	
T1	73(19.89%)	108(29.43%)	181(49.32%)	
T2	48(13.08%)	44(11.99%)	92(25.07%)	
T3	47(12.81%)	31(8.45%)	78(21.25%)	
T4	10(2.72%)	3(0.82%)	13(3.54%)	
TX	0(0.0e+0%)	1(0.27%)	1(0.27%)	
Pathologic_N				ns
N0	121 (68.0%)	128 (67.7%)	249 (67.8%)	
N1	3.00 (1.7%)	1.00 (0.5%)	4.00 (1.1%)	
NX	54.0 (30.3%)	60.0 (31.7%)	114 (31.1%)	
Pathologic_M				ns
M0	132(35.97%)	132(35.97%)	264(71.93%)	
M1	2(0.54%)	1(0.27%)	3(0.82%)	
MX	44(11.99%)	56(15.26%)	100(27.25%)	
Age				ns
Mean±SD	58.70±13.78	60.59±12.85	59.67±13.33	

Grade				*$p < 0.01$*
G1	14(3.81%)	41(11.17%)	55(14.99%)	
G2	84(22.89%)	92(25.07%)	176(47.96%)	
G3	71(19.35%)	48(13.08%)	119(32.43%)	
G4	7(1.91%)	5(1.36%)	12(3.27%)	
NA	2(0.54%)	3(0.82%)	5(1.36%)	

Median with standard deviation is shown for quantitative variables, and counts with proportions are shown for categorical variables. Abbreviations: OESG–oxidative stress- and endoplasmic reticulum stress-related gene; SD–standard deviation; ns–no significant.
